# Investigating a training supporting shared decision making (IT'S SDM 2011): study protocol for a randomized controlled trial

**DOI:** 10.1186/1745-6215-12-232

**Published:** 2011-10-26

**Authors:** Friedemann Geiger, Katrin Liethmann, Frauke Hoffmann, Jutta Paschedag, Jürgen Kasper

**Affiliations:** 1 Tumor Center, University Medical Center Schleswig-Holstein, Niemannsweg 4, 24105 Kiel, Germany; 2 Department of Pediatrics, University Medical Center Schleswig-Holstein, Schwanenweg 20, 24105 Kiel, Germany; 3 Department of Psychology, Christian-Albrechts-University Kiel, Olshausenstraße 62, 24118 Kiel, Germany; 4 Institute for Communication in Medicine - Training and Research, University of Hamburg, Martin-Luther-King-Platz 6, 20146 Hamburg, Germany; 5 Institute of Neuroimmunology and Clinical MS Research (INiMS), University Medical Center Hamburg-Eppendorf, Martinistraße 52, 20246 Hamburg, Germany; 6 MIN Faculty, Unit of Health Sciences and Education, University of Hamburg, Martin-Luther-King-Platz 6, 20146 Hamburg, Germany

## Abstract

**Background**

Shared Decision Making (SDM) is regarded as the best practice model for the communicative challenge of decision making about treatment or diagnostic options. However, randomized controlled trials focusing the effectiveness of SDM trainings are rare and existing measures of SDM are increasingly challenged by the latest research findings. This study will 1) evaluate a new physicians' communication training regarding patient involvement in terms of SDM, 2) validate SDM_MASS_, a new compound measure of SDM, and 3) evaluate the effects of SDM on the perceived quality of the decision process and on the elaboration of the decision.

**Methods**

In a multi-center randomized controlled trial with a waiting control group, 40 physicians from 7 medical fields are enrolled. Each physician contributes a sequence of four medical consultations including a diagnostic or treatment decision.

The intervention consists of two condensed video-based individual coaching sessions (15min.) supported by a manual and a DVD. The interventions alternate with three measurement points plus follow up (6 months).

Realized patient involvement is measured using the coefficient SDM_MASS _drawn from the Multifocal Approach to the Sharing in SDM (MAPPIN'SDM) which includes objective involvement, involvement as perceived by the patient, and the doctor-patient concordance regarding their judges of the involvement. For validation purposes, all three components of SDM_MASS _are supplemented by similar measures, the OPTION observer scale, the Shared Decision Making Questionnaire (SDM-Q) and the dyadic application of the Decisional Conflict Scale (DCS). Training effects are analyzed using t-tests. Spearman correlation coefficients are used to determine convergent validities, the influence of involvement (SDM_MASS_) on the perceived decision quality (DCS) and on the elaboration of the decision. The latter is operationalised by the ELAB coefficient from the UP24 (Uncertainty Profile, 24 items version).

**Discussion**

Due to the rigorous blinded randomized controlled design, the current trial promises valid and reliable results. On the one hand, we expect this condensed time-saving training to be adopted in clinical routine more likely than previous trainings. On the other hand, the exhaustivity of the MAPPIN'SDM measurement system qualifies it as a reference measure for simpler instruments and to deepen understanding of decision-making processes.

**Trial registration**

Current Controlled Trials ISRCTN78716079

## Background

### Medical decision making and the important role of communication

Good clinical practice is characterized by valuable interactions between an informed and activated patient and a proficient, proactive health care team [1]. Such interactions require a patient-health provider communication that is patient-centered, and responsive to patient needs, values, and preferences [2]. Effective communication has been associated with improvement in medical and psychological outcomes, greater patient confidence in and adherence to treatment plans, increased care satisfaction, and improved trust in health care providers [3]. Moreover, thorough patient information is an ethical obligation for the care provider [2,4]. For decisions about diagnostic or treatment options, a shared decision-making (SDM) process, where physicians and patients exchange information and perspectives is increasingly regarded as the ideal model [5]. SDM is characterized by a discussion of different options and outcomes and by the fact that patient and physician arrive at a consensus. To have unbiased and understandable information about options evidence-based patient information has been considered a prerequisite of SDM [6].

SDM is particularly suited to medical situations meeting the equipoise condition - when none of the available options appears clearly superior for an individual patient [7]. The equipoise condition is especially relevant to decisions regarding chronic illnesses with uncertain prognoses and for which treatments are only partially effective, or associated with important side-effects.

One of the most cited goals of SDM is to support medical decisions that are informed and coherent with patients' values [8]. However, the mediating principle by which SDM is hypothesized to achieve this goal has not yet been described. The method itself remains insufficiently operationalized [9,10].

### Measuring involvement

There are few instruments designed to measure SDM in clinical practice. One of the instruments most frequently used is the Observing Patient Involvement (OPTION) scale, in which an independent observer measures the extent to which clinicians initiate behavior to involve patients during the consultation [11]. This measurement strategy isn't sufficient to study clinical decision-making in its dyadic and relationship-centered approach. It is crucial to assess simultaneously patient and clinician behavior, and looking at the interaction by analysis of the fitting of communicative styles between a given patient and his physician [10,12,13]. It has been shown judging medical communication from different perspectives (physicians, patients and external observers) leads to discrepant results [14-17], and none of the existing measurement approaches can be regarded as a gold standard to assess the degree of sharing in the decision-making dyad [12,18,19]. Although the OPTION scale has provided valuable data about decision-making processes, it is only able to represent external assessments; the perceptions of participants involved in the consultation remain unavailable [20]. Based on reviews of SDM assessment instruments [12,19] a new tool, the "Multifocal approach to the sharing in SDM" (MAPPIN' SDM) has recently been developed in Hamburg (see below for details).

### The role of uncertainty in processing a medical decision and methodological implications

The decision-making process in the clinical encounter is influenced by its inherent uncertainty and by the reaction of physicians and patients to this uncertainty. Uncertainty is a feature of many aspects of physician-patient risk communication. Uncertainty is inherent in the individual prognosis and in the capacity of a treatment to improve the patient's health. Uncertainty is inherent in assessment of the physician's competence as well as in the assessment of the patient's understanding of given explanations. Uncertainty is also inherent in the interpretation of statistics, especially when evidence is lacking. Most of the uncertainties cannot be resolved within a conversation but nevertheless have to be managed. Abstracting from the particular decision, for instance on a prostate cancer screening or treatment for relapses of multiple sclerosis, we define uncertainty as the core content of the decisional communication. Uncertainty is referred to in some of the SDM literature [7,21,22]. For instance, reducing awareness of uncertainty is seen as an important goal of risk communication by some authors [23]. Given the context of uncertainty, decisional conflict is one of the key elements in decision making in clinical settings [24]. "Decisional conflict is defined as an individual perception of uncertainty about which course of action to take when choice among competing options involves risk, loss, regret, or challenge to personal life values. In lay terms, decisional conflict refers to the level of comfort that an individual experiences when facing a difficult decision. Decisional conflict should not be confused with the uncertainty inherent in the nature of the available scientific evidence. Until recently, decisional conflict in patients as well as in physicians had been studied at the level of the individual rather than at the level of the dyad, thus ignoring the interpersonal system that is at play. Individuals involved in dyadic interactions, even brief ones, can, and often do, influence each other's cognitions, emotions, and behaviours" [25]. Beyond this more quantitative approach to perception of uncertainty the methodological concept of SDM has to specify a strategy to communicate uncertainty [26]. Such a strategy might mean support for a patient, since awareness of various uncertain aspects in a decision prevents the patient from achieving a position of clarity. Negotiation of uncertainties in the medical encounter should lead to changes in the patient's cognitive representation of uncertain aspects. Beyond increasing or reducing uncertainty in the perception of the person concerned, such negotiation can change the state of the cognitive representation. There is a need for a theory about people's perception of decisional uncertainty and about the way it changes when they elaborate information relevant for this decision [27].

## Objectives

1. To evaluate a new intervention's ability to enhance physicians' communication behavior and to improve the communication in terms of SDM.

2. To further validate a newly deduced coefficient expressing the degree to which a communication involved physician and patient in an evidence-based decision making process as the gold standard for measuring SDM.

3. To evaluate SDM regarding its effects on patients' decisional conflict and internal processes of elaboration.

### Sub goals

- To further validate the short version of the Uncertainty Profile questionnaire (UP24) which is supposed to assess an individual's perception of uncertain aspects when facing a decision.

- To yield data on the interrelatedness of different perspectives on communication.

The project builds up on own previous studies and is structured to address all three study questions within one clinical trial.

## Methods

### Trial Design

**Figure 1:**
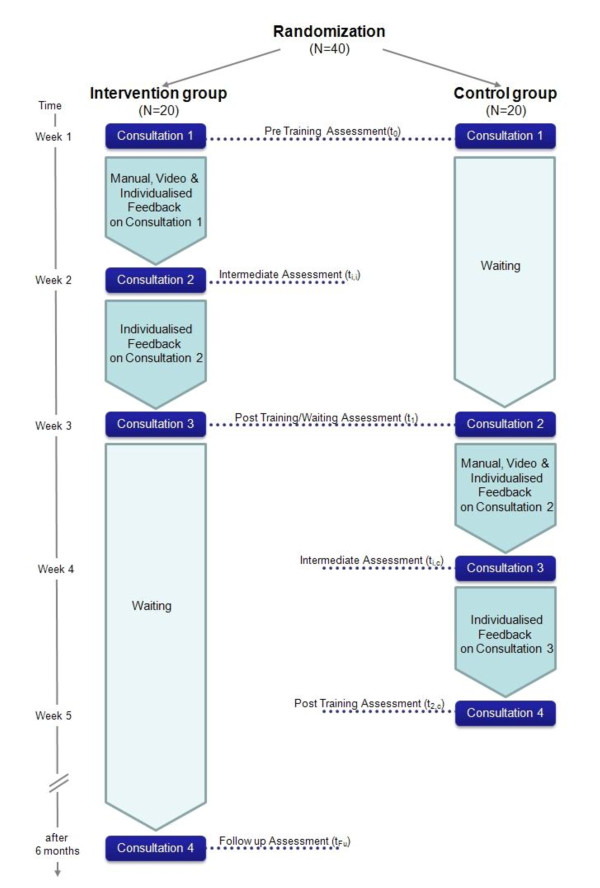
**Flow of randomly assigned participants in the two experimental conditions**.

This is a multi-center randomized controlled trial comparing a group of physicians which receives training with a waiting control group regarding the SDM performance of the doctor-patient-dyad. Within the total pool of consultations, in both groups the impact of the actual communication level according to SDM on indicators of thorough elaboration of the decision will be investigated (see Figure 1).

### Participants

As the study sample medical consultations between physicians and patients will be monitored in seven university outpatient clinics in Germany (oncology, gynecology, psychiatry, neurology, dentistry, radiology). Consultations are eligible if the physician expects a medical decision to be negotiated within the encounter. Patients are included in the study only one time, while physicians document a sequence of four consultations each.

### Intervention

The intervention is a training curriculum (doktormitSDM [28]) addressing the doctors and goaling (intending) at stimulating efforts to involve their patients in the decision making process. It has been developed based on the newest available scientific knowledge on evidence-based patient information (EBPI) and SDM [29,30]. Its didactic is inspired by training techniques from psychotherapy education and enables the participants to incorporate the theoretical framework of reference in their cognitive system by use of examples drawn from own communication behavior. Therefore, the training both coaches the physicians as observers of communication and provides feedback to them.

In particular, the training includes 3 educational components:

*i1. The manual: *It was developed to comprehensively explain background and idea of SDM as well as a set of 15 SDM skills which represent an extension of the range of skills published by Elwyn et al. [11,31]. Within the manual examples are used to illustrate varying degrees of performance.

*i2. The training video: *Corresponding with the manual a training video was developed and produced. It demonstrates excellent performance of each of the 15 indicators applied to a decision on immunotherapy in multiple sclerosis. The video shows a neurologist talking to a patient. The consultation is role play based on a prepared script. Within the video, each skill is verbally edited according to the MAPPIN'SDM framework.

*i3. The face to face feedback: *Trainees get structured feedback based on an assessment of a video-recorded own consultation in terms of the same framework of reference. The feedback session lasts a maximum of 15 minutes and follows a guideline passing 6 separate steps:

1. intro via surveying subjective benefit of the previous training steps

2. statement that feedback does not refer to communication performance in general, but just to our specific viewpoint. The trainer indicates, that there are a lot of other important aspects of communication, one could focus on. The present focus is the way the physician involves the patient into the decision to be made.

3. actualization of the specific consultation. Reporting context or particular events. e.g. by presenting a sequence of the video. Noting subject of the decision and duration of the decision sequence

4. concrete positive feedback referring to observable skills supported by presentation of video sequences.

5. investigation of the doctor's ideas regarding potential for improvement

6. suggestions regarding potential improvement. Concrete examples are provided which as far as possible build up on existing competencies. Checking understanding. Reassuring tolerable volume of input.

The training deliberately abandons to provide the trainees with a general judgment of their performance, or to reach completeness in the feedback of the communication skills. Furthermore, the feedback is not intended to go beyond the concrete consultation for instance by relating it to other consultations. The feedback aims at concreteness and traceability.

### Outcomes

As it is considered important that the trial does not negatively affect the practice process by burdening physicians and patients too much, measurement is limited to the necessary amount of instruments. Most of the measures were tested in previous studies to explore item properties and validity. In particular measures were selected to assess:

a. both parties' mutual involvement in the decision making process using the MAPPIN'SDM inventory (objective judgment based on video-tapes, physician and patient questionnaires (15 items each))

b. the patients' perception of SDM using the SDM-Q (9 items)

c. to which extent involvement (in terms of SDM) impacts on the dyadic perception of decisional conflict using the Decisional Conflict scale (16 items to be administered by physicians and patients)

d. patients' cognitive representation of decision related uncertainty using the UP24 (24 items to be administered by the patient before and after the consultation).

#### Measuring involvement by use of the MAPPIN'SDM approach

MAPPIN'SDM [32] is a measurement system comprising 7 coherent views on SDM: I) SDM related behavior shown by 1) patient, 2) doctor, 3) the doctor-patient-dyad, judged by an independent observer on the basis of video documents; II) the doctor's perception of 4) the dyad's SDM related behavior and 5) its perceived result; III) the patient's perception of 6) the dyad's SDM related behavior and 7) its perceived result. Each focus is assessed by the identical set of 15 criteria with slightly adapted formulations. This set incorporates all items of the OPTION scale [11,31] plus 4 indicators considered essential in the SDM literature.

While the complete system with 7 foci was initially developed to consequently map every possible view on SDM and thereby their interrelatedness [32], SDM can be sufficiently defined by a combination of 3 noteworthy parts. We call a decision making process "shared" if a) SDM behavior is shown by the doctor-patient-dyad (focus 3), b) the patient gets the impression of being involved (focus 7), and c) doctor and patient *share* this impression (concordance between foci 7 and 5). The formula below shows how the 3 parts are adjusted and combined to a compound measure called SDM_MASS _(SDMmeeting its concept's assumptions). SDM_MASS _ranges from 0 to 1, where 0 indicates no SDM and 1 indicates perfect SDM.

$${\text{SDM}}_{\text{MASS}}=\frac{\frac{\text{M(MAPPIN}-{\text{Qp}}_{\text{patient}})}{4}+\frac{\text{M(MAPPIN}-{\text{O}}_{\text{dyad}})}{4}+\tau_{1-15}(\text{MAPPIN}-{\text{Q}}_{\text{patient}};\text{MAPPIN}-{\text{Q}}_{\text{doctor}})}{3}$$

MAPPIN - Q_patient_: Patient questionnaire addressing his/her perceived result of SDM

MAPPIN - O_dyad_: Observer rating addressing the realized amount of SDM on the dyadic level

MAPPIN - Q_doctor_: Physician questionnaire addressing his/her perceived result of SDM

M: Mean of items of the particular domain (range 0-4)

τ_1-15_: Kendall's tau coefficient indicating consensus (concordance) on items 1-15; negative values are fixed at 0.

SDM_MASS _serves as primary endpoint for the evaluation of the training (study question 1). Accordingly, in the present study each consultation is measured using the three observer foci (MAPPIN-O_doctor _, MAPPIN-O_patient _, MAPPIN-O_dyad_) based on video data. Observation based analyses (3 × 15 items = 45 items per consultation) will be conducted by two independently working trained MAPPIN'SDM raters with previously proven inter-rater-agreement. Doctors and patients each judge the perceived SDM result by use of the MAPPIN-Q_patient _/MAPPIN-Q_doctor _after the consultation. Before filling in the questionnaires both parties shall cooperatively define the specific medical decision they will refer to. Completing these questionnaires requires about 5 minutes.

In addition, OPTION scores will be extracted from the observer data. OPTION serves as secondary endpoint of study question 1 and is used to validate MAPPIN-O_dyad _(study question 2).

#### Measuring involvement of the patient using the Shared Decision Making Questionnaire (SDM-Q)

The Shared Decision Making Questionnaire [33] assesses patients' view of a consultation. The questionnaire follows the same taxonomy of decision making as the OPTION scale and was developed to show the extent to which patients feel they were involved in the process. In its revised form SDM-Q has 9 items scoring from 0 to 3 on a 4 point Likert scale [34]. Filling in the SDM-Q requires about 5 minutes.

In the present trial, the SDM-Q will be used to validate the first component (MAPPIN-Q_patient_) of SDM_MASS _(study question 2).

In order to limit the overall number of items, SDM-Q will be assessed only in those centers where the UP24 is not applicable (see below).

#### Measuring effects of involvement using the Decisional conflict scale (DCS)

Decisional conflict defined as above can be measured by the Decisional Conflict Scale [21,35]. The DCS is a multidimensional scale of 16 items divided into 5 subscales: personal uncertainty (3 items) and its modifiable deficits of feeling uninformed (3 items), unclear values (3 items), inadequate support (3 items), and perception that an ineffective choice has been made (4 items). Meanwhile the DCS exists as a dyadic measure [36]. This application form of the DCS provides analogue items for patients and physicians. Completing the DCS requires about 5 minutes.

In the present trial, the survey of DCS on both sides (DCS_doctor_, DCS_patient_) of the doctor-patient-dyad provides the opportunity to focus two kinds of parameters: 1) the mean decisional conflict on total and single scale level viewed by the physician and by the patient and 2) the concordance of DCS ratings indicating the extent to which elaboration of the decision took place on an interpersonal level (in terms of SDM). The latter focus, operationalized by the median of Kendall's tau coefficient for the five subscales, will be used for the validation of the concordance parameter in SDM_MASS _(study question 2). The absolute DCS_patient _score in relation to the actual level of SDM serves as primary endpoint for the evaluation of SDM effects (study question 3). DCS_doctor _and DCS_patient _are assessed after each consultation.

#### Measuring effects of involvement using the Uncertainty Profile questionnaire (UP24)

The Uncertainty Profile questionnaire (formerly known as QUiCC) measures the way decision related uncertainty is represented within the patient's cognitive system and displays the intensity and profile of the patient's perception [37,38]. It assesses decisional uncertainty in eight distinct empirically grounded categories.

Corresponding to the original Uncertainty Profile questionnaire with 41 items, a short version (UP24) was developed. Each category is represented by three items. The UP24 shows good psychometric quality regarding validity, internal consitency and retest reliability. Filling in the UP24 requires about 10 minutes [39].

The UP24 can be analysed on two levels: 1) a measure of the level of uncertainty that is perceived and 2) a measure of elaboration of a decision that is indicated by the degree of differentiation between qualities of uncertainty. The latter is addressed by study question 3. For assessment of elaboration of the decision, a special coefficient (called ELAB) was invented (see formula below). Differentiation itself is operationalized by inter-scale variance. To eliminate biases caused by varying global levels of uncertainty due to individual cognitive and coping styles, each scale value is adjusted by each patient's individual grand mean over all eight uncertainty scales. Subtracting 1 from these adjusted mean scale values leads to positive values for scales above the average and negative values for those below without affecting the inter-scale variance [38]. In the present study, UP24 is administered before and after each consultation. The increase of elaboration (ΔELAB: difference between both measurement points) in relation to the actual level of SDM serves as secondary endpoint of study question 3.

The use of UP24 will be limited to consultations with ill patients (in contrast to people considering e.g. a screening). The UP24 is not used in the entire study population hence.

$$ELAB=Var\left[\left(\frac{M_{1}}{M_{total}}-1\right),\left(\frac{M_{2}}{M_{total}}-1\right)\cdots \left(\frac{M_{8}}{M_{total}}-1\right)\right]$$

Var: Variance

M_1-8_: Mean scale values of the eight uncertainty scales

M_total_: Each person's individual grand mean of uncertainty scales

#### Recording of additional information

To yield sufficient description of the study sample, diagnosis, sex and age of the patients will be recorded by the physician. Furthermore, profession, years of practical experience, age and sex of the physician will be registered. Both physician and patient have to name the decision (or its deferment) that has been made in the consultation.

### Randomization and Blinding

Randomization will be conducted by an independent person administering randomization lists (block randomization within each study center). Investigators involved in enrollment of physicians into the trial have no access to the randomization list. Concealment of allocation towards the participating physicians is considered. Physicians are informed about being randomized to one of two study arms, within which components of the intervention are provided in different order. It is emphasized, however, that both groups receive the same training modules. Observation based judgments of the dyad's SDM performance are done by two independently working raters in random order. The video documents to be judged do not include information about study arm affiliation or training stage. Thus these ratings are blinded.

### Statistical Methods

Success of randomization is checked using baseline scores of the primary endpoint SDM_MASS_, age and sex as well as practice experience of the physicians. Training effects in terms of differences between baseline and post intervention assessment are analyzed using Student's t-test. This procedure takes possible baseline imbalances regarding SDM_MASS _into account. However, if significant imbalances occurred, their influence on validity had to be discussed. Spearman correlation coefficients are used to determine convergent validities, the influence of involvement (SDM_MASS_) on the perceived decision quality (DCS) and on the increase of elaboration of the decision. The latter is operationalised by the ELAB coefficient from the UP24 (Uncertainty Profile, 24 items version). Possible cluster effects within the study centers or physicians which may mask correlations or produce artificial ones will be detected by analyzing grouped scatter plots.

### Sample Size

The calculation of the required sample size is primarily oriented towards the demands of study question 1 which addresses the evaluation of the training.

We aim at a power of 85%. Given an effect size of d = .9 (which was already realized with a former version of the training), a one-tailed t-test for two independent samples with an alpha of .05, a total sample size of N = 36 is needed. In order to take possible drop-outs into account, the sample size is expanded to N = 40.

Regarding study question 2 (validation of the components of SDM_MASS_), a number of N = 36 physicians (minimum after possible drop-outs) leads to 4 × 36 = 144 consultations in the whole study. Given alpha = .05, power = .85, this sample size would require correlations of .22 between the components of SDM_MASS _and the external criteria OPTION and DCS_concordance_. For the validation of the patient component by use of SDM-Q, only N = 76 consultations are available since the remaining complete the UP24 instead. This demands a correlation of at least .30 to yield significant results. Both these values are far below our expectations.

Regarding study question 3, correlation between SDM_MASS _and DCS_patient _has to exceed .22 (N = 144). Correlation between SDM_MASS _and ΔELAB has to go beyond .31 (N = 68). Both is regarded as achievable.

Summing up, determining sample size according to study question 1 leads to sufficient power regarding the remaining study questions.

## Discussion

This trial introduces a rigorous double-blinded randomized controlled study design. All measures and the intervention itself are either widely validated internationally and/or thoroughly tested in preliminary studies of our group. Compared to other trainings that have proven effective for training physicians in SDM, the doktormitSDM intervention is highly condensed, specific and time-saving [30,40]. This lowers the stakes of implementation as lack of time or motivation often seem to prevent wide adoption among physicians [41]. The application of the new coefficient SDM_MASS _may be too complex for some purposes. Typically, either questionnaires (from only one perspective) or observer instruments are used to assess SDM. We rather see SDM_MASS _as a gold standard which may serve as a reference measure to be used to validate and calibrate other instruments which may be less precise and comprehensive but providing higher practicability. So far, no other existing measure combines patient's, physician's and observer's view in one single measure being deduced from a coherent measurement system which is based on thorough theoretical considerations [18]. Therefore we assume this trial to inform the debate on SDM interventions as well as on SDM measurement.

Nevertheless the trial has some limitations. First, the condensed structure of the intervention which has been chosen for economic reasons in this trial may in turn lead to smaller intervention effects. Second, participation of the study centers in general and of the physicians in particular is voluntary. This may produce a selection bias towards highly motivated physicians producing non-representative consultations for analysis. Although randomization to study arms prevents major threats of validity, the results might overestimate the effect of the intervention when transferred to the average physician. Third, the sample is neither homogeneous regarding medical discipline nor a representative cross section of physicians. Further studies have to figure out generalisability of the results as well as characteristics specific to certain indications. Fourth, allocation concealment may be undermined if physicians from different groups exchange information about their schedules. However, the control group receives the complete training with only a short delay (1-2 weeks) which makes it unlikely that serious frustration influences performance in this group.

### Trial Status

Recruitment of participants started in June 2011 and is expected to end in June 2012.

## Competing interests

The authors declare that they have no competing interests.

## Authors' contributions

All authors contributed to the design and development of the study protocol. FG & JK were responsible for writing this manuscript. All authors read and approved the final manuscript.
